# COVID-19 pneumonia and ominous methemoglobinemia: Causation or confounding

**DOI:** 10.1016/j.idcr.2023.e01705

**Published:** 2023-02-02

**Authors:** Wael Goravey, Gawahir A. Ali

**Affiliations:** Department of Infectious Diseases, Communicable Diseases Centre, Hamad Medical Corporation, Doha, Qatar

**Keywords:** COVID-19 pneumonia, Methemoglobinemia, Methylene blue

## Introduction

Severe COVID-19 pneumonia and methemoglobinemia share many presenting clinical features, and it is unclear whether this may have been predominantly due to a dramatic increase in the use of oxidizing medications, which were used to treat COVID-19 during the pandemic, or COVID-19 itself. Initial correct assessment is sometimes difficult, particularly if the clinical judgment has been confounded towards the emerging disease [1]. To highlight such a dilemma, we report a case of COVID-19 pneumonia and concomitant significant methemoglobinemia to raise awareness of the emergence of methemoglobinemia amidst the COVID-19 pandemic.

## Case description

A 30-year-old gentleman presented with a two-day history of increasing shortness of breath and headache. He was in the isolation facility due to COVID-19 pneumonia diagnosed 5 days before this event. He was not on any medications, particularly hydroxychloroquine. He had no significant medical history.

**Fig. 1:**
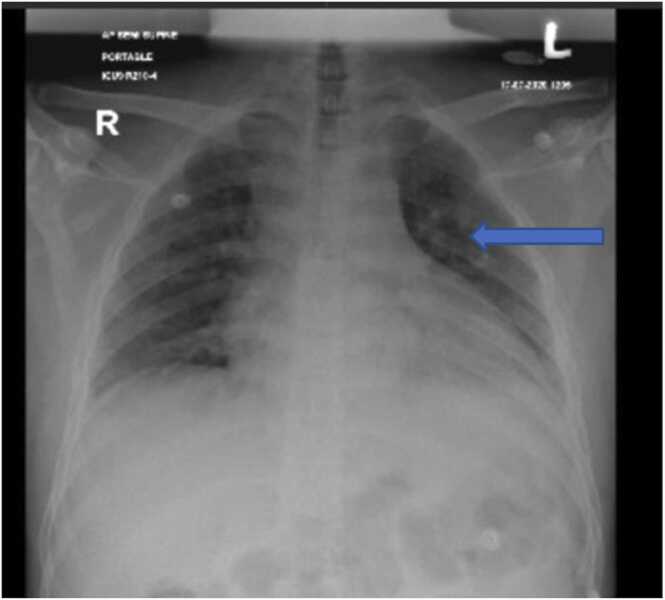
Anterior Posterior (AP) view of chest X ray. Accentuation of the Broncho-vascular markings in the left middle lung zone (Blue arrow).

**Fig. 2:**
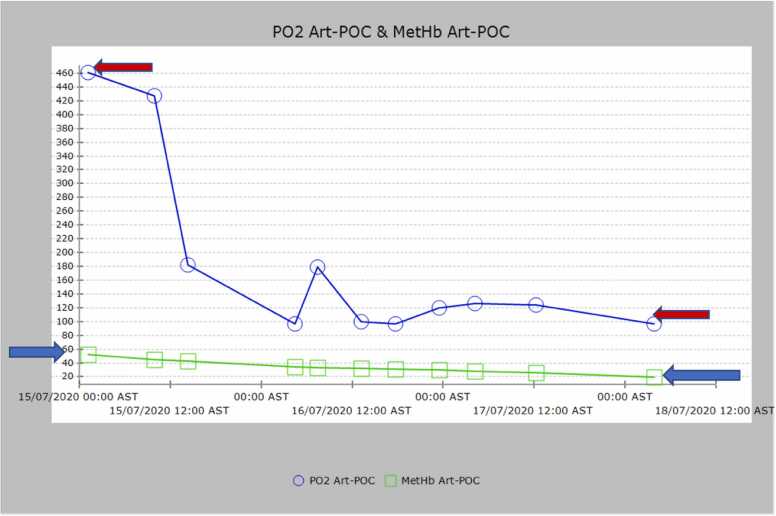
ABG. Partial pressure of oxygen (Blue line) and methemoglobinemia level (Green line) on admission (460 mmHg; FiO2 31 %, with MetHb level of 55 %, Red and blue arrows) and steady improvement with management (100 mmHg, FiO2 21 %, vs MetHb level of 3 %).

**Fig. 3:**
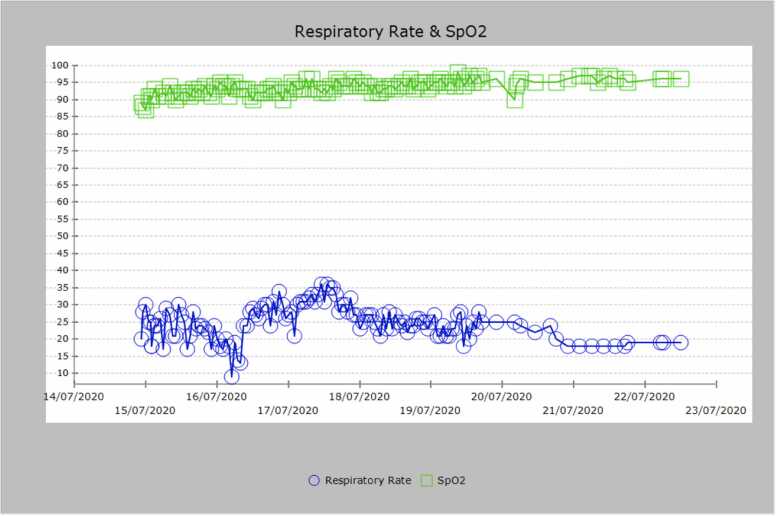
Oxygen saturation (Green line) with respiratory rate (Blue line) on admission and follow up.

Examination showed, afebrile, tachypnea with a respiratory rate of 35 breaths per minute, oxygen saturation of 85 % on room air, bibasal crackles, and unremarkable other findings. Thus, the progression of his COVID-19 pneumonia or pulmonary embolism was suspected. However, his CXR revealed a stable broncho-vascular marking in the left middle lung zone (Fig. 1). Given worsening respiratory status and a discrepancy in the oxygen saturation (PO2 460 mmHg; FiO2 31 %, vs Spo2 85 %), methemoglobinemia was suspected and confirmed with a level of 55 % (Table 1). He was commenced on parenteral methylene blue, ascorbic acid and supplemental oxygen with complete resolution of his symptoms and normalization of methemoglobin level (Fig. 2, Fig. 3).

**Table 1:** Laboratories test.

Test	Value
Hemoglobin level	14 g/dL (Normal range 13.5–16.6)
White blood cell count	5.6 × 10^9^/L (Normal range 4–11)
C-reactive protein (CRP)	6 mg/l (Normal range 0–5)
Spo2	85 %
pH	7.40(Normal range 7.35–7.45)
PO2 mmHg, FiO2 31 %	461 mmHg
PCO2 mmHg	31 mmHg
Methemoglobin level (MetHb)	55 % (Normal is less than 1 %)
Glucose-6-phosphate dehydrogenase level(G6PD)	Normal level
Hb electrophoresis	Normal Hb pattern
Cytochrome b5 reductase level	Normal level

## Discussion

With the evolving COVID-19 pandemic, a range of hematologic findings and complications were described including methemoglobinemia [2]. Antimalarial agents, particularly hydroxychloroquine have been associated with methemoglobinemia however, given the range of atypical presentation of this pandemic and the absence of a plausible explanation of methemoglobinemia in our case, COVID-19-induced methemoglobinemia worth to be considered awaiting more evidence [3].

## CRediT authorship contribution statement

**WG:** Corresponding author, Clinical management, contribute to data acquisition, manuscript preparation and final proof reading.

**GA:** Data acquisition, designed the tables and figures, performed critical review and manuscript writing.

## Funding

No funding was received towards the publication.

## Ethical approval

Ethics approval and permission was obtained to publish the case reports from the institutional review board which is in line with international standards.

## Consent

A written informed consent was obtained from the patient to include clinical presentation together with results and imaging. This was subsequently reviewed and approved by the institution ethics and research review board with MRC-04-22-425.

## Conflict of interest

The authors declare that they have no competing interests.

## Data Availability

The authors confirm that the datasets supporting the findings of this case are available from the corresponding author upon request.
